# A novel web-based 24-h dietary recall tool in line with the Nova food processing classification: description and evaluation

**DOI:** 10.1017/S1368980023001623

**Published:** 2023-10

**Authors:** Daniela Neri, Kamila Tiemann Gabe, Caroline Dos Santos Costa, Euridice Martinez Steele, Fernanda Rauber, Dirce Maria Marchioni, Maria Laura da Costa Louzada, Renata Bertazzi Levy, Carlos Augusto Monteiro

**Affiliations:** 1 Center for Epidemiological Studies in Health and Nutrition, University of Sao Paulo (Nupens/USP), Sao Paulo, Brazil; 2 Department of Nutrition, School of Public Health, University of São Paulo, Sao Paulo, Brazil; 3 Department of Preventive Medicine, School of Medicine, University of Sao Paulo, Sao Paulo, Brazil

**Keywords:** Comparative study, Dietary assessment, web-based self-completed 24-h recall, Ultra-processed foods, Nova food classification system

## Abstract

**Objective:**

This paper describes the first web-based self-completed 24-h recall designed to categorise food intake according to Nova groups – Nova24h – and its agreement with a reference tool in estimating the dietary relative contribution of the four Nova food groups (% of total energy intake).

**Design:**

Comparisons of estimates of dietary relative contributions of Nova groups obtained by Nova24h and one standard interviewer-led 24-h recall.

**Setting:**

Nationwide adult cohort study in Brazil.

**Participants:**

The subjects were 186 participants of the NutriNet Brasil Cohort Study (*n* 186).

**Results:**

No statistically significant differences were observed between the Nova24h and the reference tool mean contributions of unprocessed or minimally processed foods (52·3 % *v*. 52·6 %), processed culinary ingredients (11·6 % *v*. 11·9 %), processed foods (17·1 % *v*. 14·7 %) and ultra-processed foods (19·0 % *v*. 20·9 %). Intraclass correlation coefficients between individual estimates obtained for each Nova group showed moderate to good agreement (0·54–0·78). Substantial or almost perfect agreement between the tools was seen regarding the ability to rank participants according to quintiles of contribution of each Nova group (PABAK 0·69–0·81).

**Conclusions:**

Nova24h is a suitable tool for estimating the dietary relative energy contribution of Nova food groups in the NutriNet Brasil cohort. New studies are necessary to verify its adequacy in other populations.

The Nova food classification is a system that categorises foods based on the extent and purpose of food processing they undergo^(1)^. Many studies worldwide have used the Nova system to assess diet and health relationships^(2–7)^ nurturing its further use as a framework for national dietary guidelines and dietary guidance from national and international health associations^(8)^. This growing interest in food processing has prompted researchers to explore methods for measuring the extent of processing of dietary data.

Some strategies have been proposed to determine the level of processing of foods in large studies and national surveys collected through widely used standard dietary assessment tools, such as interviewer-led 24-recall and FFQ^(9)^, often lacking details about food processing. Additionally, new tools for collecting dietary data specifically designed to discriminate foods according to the level of food processing have been developed^(10,11)^.

The 24-h multiple-pass dietary recall applied by trained dietitians is considered a reference method among dietary assessment tools for collecting quantitative data regarding both absolute and relative food group or nutrient dietary intakes^(12)^. It captures detailed dietary information, as interviewers ask individuals to recall and inform in detail all foods and drinks they consumed over the last 24 h^(13)^. However, this tool is often expensive and labour intensive for researchers and time consuming for study participants^(14)^.

Recently developed, validated, and available in a few countries, web-based self-completed 24-h dietary recall tools offer a low-burden and cost-effective alternative for collecting dietary data. These include the INTAKE24^(15)^ and the Oxford WebQ^(16)^ in the UK, the Automated Self-Administered 24-H Dietary Assessment Tool (ASA24)^(17)^, available in Australia, Canada and the US, and the tool used by the NutriNet Santé Cohort Study in France^(18)^. These tools can be used in substitution to the 24-h multiple-pass dietary recall to obtain dietary intake data and estimate energy and nutrients intakes.

The Nova 24-h dietary recall (Nova24h) is the first web-based self-completed tool that collects 24-h food intake data in line with the Nova system. It automatically classifies every food item into one of the four Nova groups: *unprocessed or minimally processed foods*, *processed culinary ingredients*, *processed foods* and *ultra-processed foods*. This tool was developed to be used in the NutriNet Brasil Cohort Study launched in January 2020 to investigate prospective associations between dietary patterns and chronic non-communicable diseases^(19)^.

The objective of the present study is to describe the development of the Nova24h tool and to evaluate its agreement with a standard interviewer-led multiple-pass 24-h dietary recall in estimating the dietary relative energy contribution of each of the four groups as defined by the Nova classification system among participants of the NutriNet Brasil cohort.

## Methods

### Development of Nova24h

**Fig. 1:**
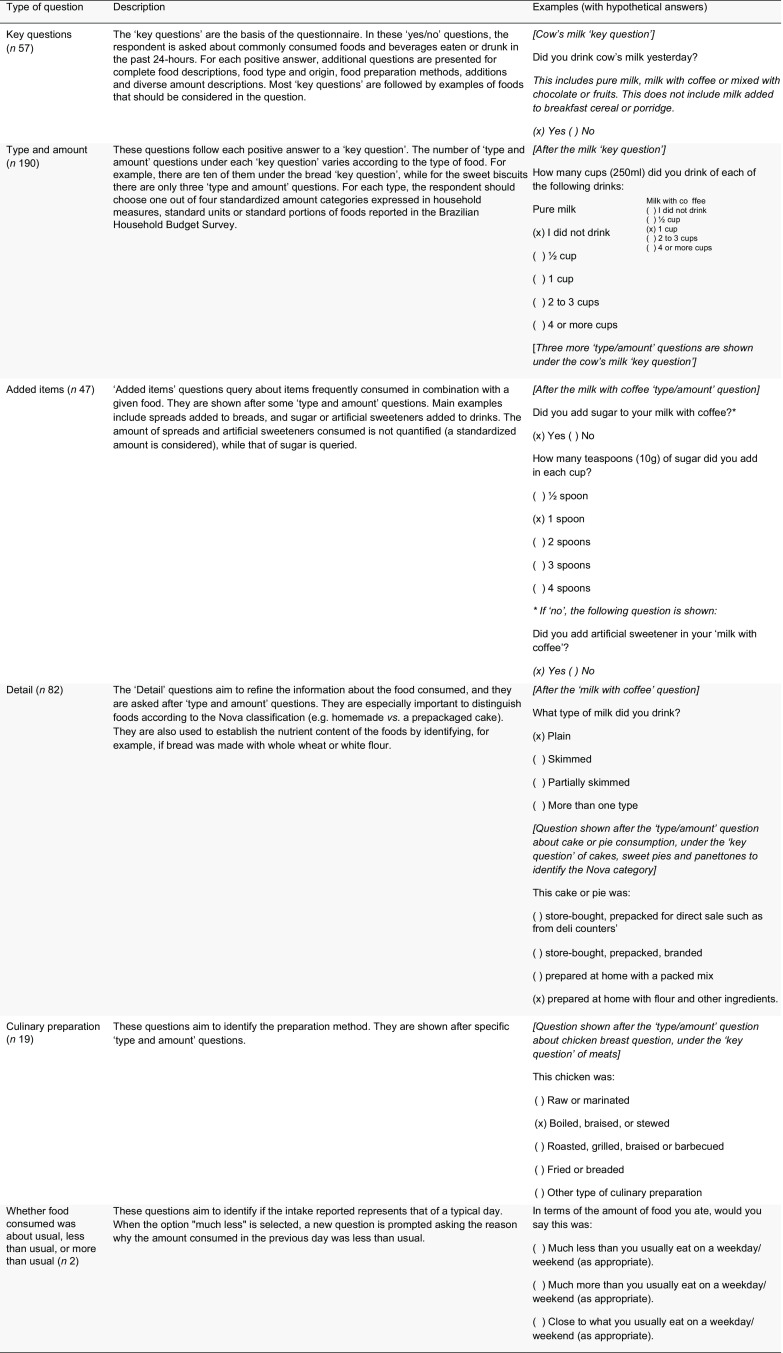
Structure of the Nova24h tool. An illustration of the types of questions asked in the Nova24h tool, their description, and examples with hypothetical answers

Nova24h web-based self-completed tool was designed by a team of nutrition epidemiologists from the same research center at the University of Sao Paulo that developed the Nova food classification system. It consists of a series of 395 concatenated close-ended questions about all foods and drinks consumed during the previous day, and two questions about whether the overall amount of food consumed during the previous day was about usual, less than usual or more than usual – as described in Fig. 1. The food list incorporated into the Nova24h was developed based on foods reported by participants of one nationally representative dietary survey conducted in Brazil in 2008–2009^(20)^.

All questions are answered with touches on the mobile screen or clicks on the computer. To enhance the usability of the program, large radio buttons and simple scrolling fields were included. The format of the questionnaire and the structure of the questions were based on feedback from extensive piloting of the tool by researchers from the Center for Epidemiological Research in Health and Nutrition at the University of Sao Paulo. The Nova24h estimated completion time is about 15 min.

Before initiating the recall, participants are provided with brief instructions on how to complete the Nova24h questionnaire. They are then asked a question related to food restrictions or special diet (e.g. lactose-, gluten- and/or red meat-free, vegetarian or vegan) and redirected to the questions about food items consumed in the previous day. Participants are presented with 57 ‘yes/no’ key questions about commonly consumed foods and drinks (e.g. ‘Did you eat fish yesterday?’). For positive answers, subsequent questions are prompted to specify the type (e.g. fresh fishes such as salmon, tuna, sardine, hake and tilapia and salted fishes, such as cod or canned fish) and amount consumed of each selected food item (e.g. number of fish filets consumed) (*n* 190).

The questions were designed to reduce respondent burden by optimising the number of food items covered by each question. In this sense, each question may refer to one (e.g. cereal bar) or more than one related individual items/culinary preparations (e.g. 1. fruit compote, guava paste, pumpkin jam or marmalade; 2. pudding, manjar or mousse and 3. coxinha, pie, sfiha and kebab). Amounts are reported using standardised categories, including portions of commonly used household measures (e.g. bowls and spoons) and standard units (e.g. an apple, a can)^(20)^.

Certain ‘type and amount’ answers also prompt questions about added items (e.g. sugar added to coffee) (*n* 47), preparation method in the case of home-made dishes (e.g. raw/marinated, cooked, sautéed or stewed, roasted, grilled, barbecued, fried, breaded, in case of fish) (*n* 19) and further details to refine the information about the food consumed (e.g. homemade or purchased ready-to-eat, in case of cakes) (*n* 82).

To avoid the problem of respondents recording the same food item more than once under different questions, warning messages were added throughout the recall process. For instance, the question ‘Did you drink milk yesterday?’ is followed by the warning: *Attention! DO NOT include milk added to porridge or breakfast cereal here so as not to duplicate what you ate. If that’s the case, change your answer above to ‘no’*. After reporting all foods and drinks consumed during the previous day, participants are asked two questions to determine whether the previous day’s total intake represents his/her’s typical daily intake. After completing the recall, participants are asked an additional question regarding use of vitamin or mineral supplements.

A description of the food items and all their possible variations within each of the fifty-seven key questions is presented in the online Supplementary Table S1. A total of 526 food items capture all possible combinations of responses to questions within each of the fifty-seven key questions, including 347 individual or grouped items (e.g. ‘whole milk’ or ‘squash, zucchini or eggplant’) and 179 culinary preparations, which are subsequently disaggregated into underlying ingredients (e.g. ‘cooked rice’ is disaggregated into: rice, oil, onion, garlic and salt). Standardised recipes from the *Tabela Brasileira de Composição de Alimentos* 7·0^(21)^ (TBCA – Brazilian Table of Food Composition) were the primary source used for the disaggregation. When a standardised recipe for some specific dish was not available in the TBCA (*n* 10), an adapted TBCA recipe was used to attribute ingredients and their respective proportions.

The nutritional composition of each individual or grouped food items and ingredients from the culinary preparations was estimated using food codes from the TBCA. Food codes from the United States Department of Agriculture^(22)^ National Nutrient Database were used to code foods with no match with the TBCA (11 out of 526). For grouped food items, the food code representing the most consumed food (according to the national survey^(20)^) was used (e.g. ‘zucchini’ food code was used for coding ‘squash, zucchini or egg-plant’).

A three-stage process was undertaken to classify the 347 food items and ingredients of 179 culinary preparations according to the extent and purpose of industrial food processing as established by the Nova food classification system. First, two researchers working independently (E.M.S and C.S.C) assigned food items and ingredients to one of four mutually exclusive Nova groups and subgroups (online Supplementary Table S2). Second, Nova food groups and subgroups data were reviewed independently by two separate researchers (D.N and K.G). Food items and ingredients for which there was consensus in the categorisation among all researchers were assigned to their Nova group and subgroup. Food items with disagreement in categorisation between any two researchers were shortlisted and flagged for further scrutiny. At stage three, an expert panel of two nutrition epidemiologists (R.B.L and M.L.L) with substantial experience working with the dietary intake in the national dietary survey was convened to review, discuss and reach an agreement about the categorisation of the short-listed products.

A data set in a long format including the 526 food items and the underlying ingredients of culinary recipes, as well as their NOVA classification, food codes and nutritional composition, was built into the system. Using this matrix, Nova24h automatically generates an output informing all the foods and amounts consumed by the respondents with their respective nutritional content and classification according to Nova. Though the Nova24h was designed to estimate the energy and nutrient contents of the diet, its ability to do so in comparison to a standard tool has not yet been tested.

### Evaluation of the agreement between Nova24h and the standard method

#### Study sample

All participants of the NutriNet Brasil Cohort Study who completed the Nova24h tool between 18 September and 16 October 2020 were consulted in the online platform about whether they would accept to participate in the agreement study. Among those who agreed to participate (nearly 3/4 of participants), a sample of 186 participants, selected to mimic the demographic distribution (age, sex and region of residence) of the total adult Brazilian population, was studied. A sample size of at least 152 is required for reaching 80 % power in detecting even weak agreements (intraclass correlation coefficient = 0·2) between two observations per subject^(23)^.

#### Data collection

Following completion of the Nova24h recall, the selected sample was contacted over the phone on the same day to respond to the reference method – the interviewer-led multiple-pass 24-h dietary recall^(13)^. Eighty-five percent of participants completed the two recalls on weekdays and 15 % on weekend days.

The multiple-pass interviews were carried out using the Brazilian version of the GloboDiet software^(24)^ by two dietitians skilled in the use of this tool. The dietitians were trained to pay particular attention to food intake information needed to capture the level of processing of food items. This included specific information on the preparation or processing of certain food items (e.g. home-made from scratch or ready-to-eat products), brand names of packaged products (for branded breakfast cereal and breads, for instance), the place of preparation (at restaurant, street-food, take-away places), as well as the method of preparation of mixed dishes and the types of ingredients used (e.g. from scratch with fresh ingredients or pre-made and frozen). The dietitians were blinded to what the participants had entered in the Nova24h. All food items from the GloboDiet database were coded into food codes and subsequently classified according to Nova by the same researchers (E.M.S and C.S.C) using the same procedures used in Nova24h. The same TBCA food composition table used in Nova 24h was used to calculate nutrient intakes in the GloboDiet.

### Data analysis

Standardised procedures were taken to impute or logically calculate estimations. When individuals selected a food item and its amount but did not complete data fields relating to food type (e.g. plain or skimmed), source (e.g. homemade, or packed) or preparation (e.g. roasted, or fried) (*n* 23 participants), the amount informed was distributed among all options available for each food, following the distributions observed in the first 27, 927 participants of the NutriNet Brasil Cohort Study who had completed one Nova24h. In the current analysis, for example, if a participant did not inform the origin of honey bread, 20·4 % of the reported amount was considered ‘homemade’, 28 % ‘bought at a bakery’ and 51·6 % ‘branded packed’, as these were the proportions reported by the 27, 927 participants. Quality control procedures were implemented to verify that appropriate data selection, calculation methods and data entry were used.

Descriptive statistics including mean values (and standard deviations) and frequency distribution were used to describe sample characteristics. The mean dietary contribution of each Nova group expressed in percentage of total energy intake, with 95 % confidence intervals, obtained with Nova24h was compared with the same estimates obtained with the reference method.

Intraclass correlation coefficients (ICC), derived from two-way mixed-effects models, were used to assess the strength of agreement between the methods in the overall sample. ICC across socio-demographic and weight status categories were assessed as secondary analysis. Values less than 0·50 were interpreted as indicative of *poor* agreement, between 0·50 and 0·75 as *moderate* agreement, between 0·75 and 0·90 as *good* agreement, and values greater than 0·90 as indicative of *excellent* agreement^(25)^.

Finally, to assess whether the methods agree in ranking individuals into the same or adjacent quintiles of consumption of each Nova food group, we divided participants into quintiles of the dietary contribution of each Nova group (% of total energy intake) as estimated by each method. The percentage classified into the same quintile by both methods was calculated and prevalence-adjusted and bias-adjusted kappa (PABAK) was used to evaluate the level of agreement. Values of PABAK were interpreted as follows: ≤ 0 *no agreement*, less than 0·20 *none to slight*, 0·21–0·40 *fair*, 0·41–0·60 *moderate*, 0·61–0·80 *substantial* and 0·81–1·00 indicating *almost perfect* ^(26)^.

Comparison between the two methods regarding the dietary energy contribution of subgroups within each Nova group was also performed as secondary analyses. All analyses were conducted using the STATA statistical software package version 15·0.

## Results

Socio-demographic characteristics of the study participants are shown in Table 1. Mean age was 41·3 years, 55 % were women, 61 % were from the most populous Brazilian regions (Southeast and Northeast) and 95·1 % had completed secondary school (46·2 %) or college/university (48·9 %). The mean BMI of study participants was 26·8 kg/m^2^; 30 % of participants were overweight and 24 % obese.

**Table 1:** Socio-demographic and anthropometric characteristics of study participants (*n* 186)

Characteristics	*n*	%
Age (years)		
Mean	41·3	
sd	12·0	
Sex		
Female	103	55·0
Region		
North	16	8·6
Northeast	53	28·5
Center-West	22	11·8
Southeast	60	32·3
South	35	18·8
Educational level		
Less than elementary	4	2·2
Elementary	5	2·7
Secondary	86	46·2
College/university	91	48·9
BMI status		
< 18·5 kg/m^2^	3	1·6
18·5–25 kg/m^2^	82	44·1
25–29·9 kg/m^2^	56	30·1
≥ 30 kg/m^2^	45	24·2

The contribution of Nova food groups to the total energy intake estimated with Nova24h or with the reference tool (24-h multiple-pass dietary recall) is shown in Table 2. No statistically significant differences were observed between the Nova24h and the reference tool mean contributions of unprocessed or minimally processed foods (52·3 % *v*. 52·6 %), processed culinary ingredients (11·6 % *v*. 11·9 %), processed foods (17·1 % *v*. 14·7 %) and ultra-processed foods (19·0 % *v*. 20·9 %).

**Table 2:** Dietary contribution (% of total energy intake) of nova food groups using the Nova24h tool or the interviewer-led 24-h dietary recall (reference method) (*n* 186)

	Nova24h		Reference method		Intraclass correlation coefficients	
Nova food groups	Mean	95 % CI	Mean	95 % CI	ICC	95 % CI
Unprocessed or minimally processed foods	52·3	49·9, 54·7	52·6	49·9, 55·2	0·78	0·71, 0·84
Processed culinary ingredients	11·6	10·4, 12·8	11·9	10·6, 13·2	0·54	0·38, 0·65
Processed foods	17·1	15·2, 19·0	14·7	12·7, 16·7	0·72	0·62, 0·79
Ultra-processed foods	19·0	17·0, 21·0	20·9	18·5, 23·2	0·75	0·66, 0·81

ICC, Intraclass correlation coefficients.

ICC between individual estimates obtained with each tool showed *moderate* agreement for both processed culinary ingredients (0·54; 95 % CI 0·38, 0·65) and processed foods (0·72; 95 % CI 0·62, 0·79) and *good* agreement for both unprocessed or minimally processed foods (0·78; 95 % CI 0·71, 0·84) and ultra-processed foods (0·75; 95 % CI 0·66, 0·81). ICC did not substantially change across socio-demographic or weight status categories (online Supplementary Tables S3–S6).

The mean dietary contribution of food subgroups within each Nova group estimated with Nova24h or with the reference tool and the corresponding ICC are presented in online Supplementary Table S2. *Moderate* or *good* agreement was seen for most subgroups of unprocessed or minimally processed foods, processed culinary ingredients, processed foods and ultra-processed foods.

Table 3 assesses whether the two tools agree in ranking individuals into the same or adjacent quintiles of consumption of each Nova food group and inform the corresponding PABAK. *Substantial* agreement was seen for unprocessed or minimally processed foods, processed culinary ingredients and ultra-processed foods (PABAK of 0·78, 0·69 and 0·77, respectively) and *almost perfect* agreement for processed foods (PABAK of 0·81).

**Table 3:** Agreement between participants classification according to quintiles of the dietary energy contribution of each Nova group estimated using the Nova24h and the interviewer-led 24-h dietary recall (reference tool) (*n* 186)

Quintiles (Q) estimated with the reference method*	Quintiles (Q) estimated with the Nova24h tool^†^					PABAK	
	Q1	Q2	Q3	Q4	Q5		95 % CI
Unprocessed or minimally processed foods						0·78	0·60, 0·96
Q1	11·3	5·9	1·6	1·1	0·5		
Q2	3·2	5·4	5·9	4·3	1·1		
Q3	2·7	5·4	4·3	5·4	2·2		
Q4	2·2	1·6	6·5	3·8	5·9		
Q5	1·1	1·6	1·6	5·4	10·2		
Processed culinary ingredients						0·69	0·48, 0·90
Q1	10·2	6·5	0·0	2·2	1·6		
Q2	4·3	3·8	5·9	2·7	3·2		
Q3	2·7	3·2	7·0	2·7	4·3		
Q4	1·6	2·7	3·8	7·0	4·8		
Q5	1·6	3·8	3·2	5·4	5·9		
Processed foods						0·81	0·64, 0·98
Q1	12·4	2·7	2·2	2·2	1·1		
Q2	4·3	7·0	7·0	1·6	0·0		
Q3	2·2	7·5	3·8	4·3	2·2		
Q4	1·1	2·2	3·8	7·0	5·9		
Q5	0·5	0·5	3·2	4·8	10·8		
Ultra-processed foods						0·77	0·59, 0·95
Q1	10·2	4·3	4·3	1·6	0·0		
Q2	6·5	4·8	3·2	4·3	1·1		
Q3	1·1	5·9	4·8	3·8	4·3		
Q4	2·7	3·2	4·8	3·8	5·4		
Q5	0·0	1·6	2·7	6·5	9·1		

PABAK, Prevalence-adjusted bias-adjusted kappa; Q, quintiles.

* Ranges for the dietary contribution (%) of unprocessed or minimally processed: Q1 = 1·2–36·7; Q2 = 37·0–46·1; Q3 = 46·2–58·3; Q4 = 58·3–68·2; Q5 = 68·6–98·1; of processed culinary ingredients: Q1 = 0·3–4·0; Q2 = 4·1–7·8; Q3 = 8·0–11·9; Q4 = 12·1–19·7; Q5 = 19·7–48·4; of processed foods: Q1 = 0–1·7; Q2 = 2·2–8·4; Q3 = 8·4–15·6; Q4 = 15·9–24·8; Q5 = 25·4–65·9; of ultra-processed foods: Q1 = 0–6·0; Q2 = 6·2–12·8; Q3 = 13·1–23·1; Q4 = 23·2–34·5; Q5 = 35·3–63·6.

† Ranges for the dietary contribution (%) of unprocessed or minimally processed foods: Q1 = 4·2–37·8; Q2 = 37·9–47·7; Q3 = 48·0–56·7; Q4 = 57·2–65·3; Q5 = 65·4–96·9; of processed culinary ingredients: Q1 = 0·1–4·3; Q2 = 4·4–7·9; Q3 = 8·1–11·8; Q4 = 12·0–18·0; Q5 = 18·1–37·4; of processed foods: Q1 = 0–5·7; Q2 = 5·8–12·5; Q3 = 12·7–17·6; 4 = 18·1–26·9; Q5 = 27·3–73·1; of ultra-processed foods: Q1 = 0–6·4; Q2 = 6·5–12·6; Q3 = 13·0–19·8; Q4 = 19·9–30·5; Q5 = 30·5–63·8.

## Discussion

The present study described the first web-based self-completed 24-h dietary recall tool designed to assess dietary intake in line with the Nova food classification system and evaluated the agreement between this new tool and a reference tool in estimating the dietary contribution of each of the four Nova groups in a sample of participants of the NutriNet Brasil Cohort Study.

We found that the mean dietary energy contribution of each Nova group was almost identical or very close when estimated by the Nova24h or the interviewer-led multiple-pass 24-h dietary recall tool. The agreement between the two tools in estimating participants’ individual dietary contribution of Nova groups was *moderate* for processed culinary ingredients and processed foods and *good* for unprocessed or minimally processed foods and ultra-processed foods. The agreement to rank participants according to quintiles of each Nova food group consumption was *substantial* for unprocessed or minimally processed foods, processed culinary ingredients and ultra-processed foods and *almost perfect* for processed foods.

The lower agreement between the two tools for processed culinary ingredients (ICC of 0·54 against > 0·70 for the other three Nova groups) is probably explained by the fact that, contrary to the reference tool, Nova24h does not ask participants about oils added to each preparation after they were cooked. This is confirmed by the low ICC regarding vegetable oils shown in online Supplementary Table S2 (0·31). The initial decision to not include oils added at the table was made to reduce participants’ burden, but a new updated version of Nova24h will include this question.

As other web-based self-completed dietary recall tools^(14–18)^, Nova24h has many advantages over interviewer-led recalls. It allows considerable logistic simplification and cost savings; it provides greater flexibility, allowing the subject to complete the recall at any time via a user-friendly interface. Importantly, such advantages may increase participation and retention rates in epidemiological studies. Its main limitation, also shared with similar tools, is the requirement of participants’ literacy and minimum computer skills.

Different from existing web-based self-completed dietary recall tools^(14–18)^, Nova24h was developed to address the need for accurately assessing dietary intake according to food processing levels as defined by the Nova food classification system.

Some study limitations should be noted. Although a sample size of 186 is considered enough for detecting even weak agreements^(23)^, it was not calculated to examine differences according to socio-demographic characteristics or weight status. Though weekend days were underrepresented in this comparative study sample, this will unlikely change the agreement between the tools, since any deviations from true values would probably affect both tools. Also, due to the high schooling levels of the NutriNet Brasil Cohort^(19)^, 95 % of the study participants had at least completed high-school education, a condition found only in nearly half of the Brazilian adult population^(27)^. Thus, caution is required when extrapolating the present results to the general Brazilian population or to other Brazilian populations with lower education. Adaptations to the original Nova24h recall are probably necessary for its application in populations with differing dietary patterns from those found in Brazil.

In terms of study strengths, the study design ensured that both tools collected dietary intake data for the same 24 h and analysed them using the same food composition table. In addition, the same researchers applied the Nova classification to both sets of data.

Our findings suggest that the low-cost Nova24h may be a suitable method for assessing dietary data according to food processing. Further work will include the evaluation of the performance of this new tool for estimating energy and nutrient intakes.

## Conclusion

This study indicates that Nova24h is a suitable tool for assessing dietary relative energy contribution in line with the Nova food system classification among participants of the NutriNet Brasil Cohort Study. New studies are necessary to verify the potential application of Nova24h in other populations.

## Supplementary material

For supplementary material accompanying this paper visit https://doi.org/10.1017/S1368980023001623. click here to view supplementary material
