# Supplementary material for: A novel web-based 24-h dietary recall tool in line with the Nova food processing classification: description and evaluation
Source: Public Health Nutr. 2023 Aug 7;26(10):1997–2004. doi: 10.1017/S1368980023001623 (PMC10564608; doi:10.1017/S1368980023001623)
Supplement: Supplementary file 1 [file S1368980023001623sup.zip › S1368980023001623sup001.docx]

**Supplementary Table S1. Description of food items and all their possible variations within each of the 57 key-questions.**

|  | PORTUGUES | INGLES |
| --- | --- | --- |
| 1 | Agua pura; | Plain water; |
| 2 | Café com e sem adicao de acucar; | Coffee with and without added sugar or artificial sweetener; |
| 3 | Leite vegetal ou outra bebida vegetal, com ou sem adicao de acucar, nas versões diet, light ou tradicional; | Milk or beverages alternatives, with and without added sugar, in their classical, diet or light forms; |
| 4 | Leite de vaca, incluindo leite puro, leite com café, chocolate ou fruta, com ou sem adicao de acucar, nas versões integral, desnatado ou semidesnatado; | Milk, including pure milk, milk with coffee, chocolate, and fruit, with and without added sugar or artificial sweetener, in whole, skim or low-fat versions; |
| 5 | Refrigerante nas versões diet, light ou zero; | Soft drinks, in their classical, diet, light or zero sugar forms; |
| 6 | Suco de fruta feito na hora a partir da fruta ou da polpa, com ou sem adicao de acucar ou adocante; | Freshly squeezed fruit juice made from fruit or pulp, with and without added sugar or artificial sweetener; |
| 7 | Suco de fruta industrializado, incluindo suco 100% integral ou do tipo Tang, Maguary ou outras marcas, com ou sem adicao de acucar ou adocante; | Packaged fruit juice, including “100% fruit juice” or brands like “Tang”, “Maguary” or others, with and without added sugar or artificial sweetener; |
| 8 | Iogurte natural ou iogurte com sabor, podendo ser integral ou desnatado, com ou sem adicao de acucar ou adocante; | Plain and flavored yogurt, which can be whole or low-fat, with and without added sugar or artificial sweetener; |
| 9 | Chá feito em casa, com ou sem adicao de acucar ou adocante; | Homemade tea, with and without added sugar or artificial sweetener; |
| 10 | Outras bebidas industrializadas, incluindo chá gelado, isotônico/energético ou suplemento alimentar/esportivo; | Other packaged beverages, including iced tea, sports or energy drinks, or nutritional supplements. |
| 11 | Bebidas alcoólicas, incluindo cerveja, vinho ou destilados e/ou drinks a base de destilados; | Alcoholic beverages, including beer, wine, or spirits and/or mixed drinks made with spirits; |
| 12 | Todos os tipos de pão salgado, incluindo pão francês, baguete, pão italiano, pão caseiro, pão sirio ou arabe, pão de forma, pão de cachorro quente ou hambúrguer, bisnaguinha, pão de queijo, croissant e torrada de pacote; podendo ser branco ou integral, com ou sem adição de spreads (manteiga, margarina, geléia, etc.). | All types of savory bread, including French bread, baguette, Italian bread, homemade bread, Syrian or Arabic bread, sandwich loaf, hot dog or hamburger buns, dinner rolls, cheese bread, croissant, and packaged toast bread; with and without added spreads (butter, margarine, jam, etc.). The breads may be white or whole wheat. |
| 13 | Pão doce, broa, pão de mel, "croissant" doce, sonho ou churros, podendo ter sido feito em casa, comprado em padaria/casa de bolos ou embalado com marca; | All types of sweet breads, including, *broa^^[[1]](#footnote-1)^^* , honey bread, sweet croissants, donuts, or churros. The breads might be homemade, bought from a bakery/cake shop or packaged with a brand; |
| 14 | Cuscuz de tapioca ou de milho ou tapioca/beiju, com ou sem adição de spreads (manteiga, margarina, geléia, etc.); | Tapioca or cornmeal couscous or tapioca/beiju, with and without added spreads (butter, margarine, jam, etc.); |
| 15 | Biscoito salgado, podendo ser tradicional ou integral, com ou sem adição de spreads (manteiga, margarina, geléia, etc.); | Crackers, with and without added spreads (butter, margarine, jam, etc.). The crackers may be white or whole wheat; |
| 16 | Biscoito doce ou cookies, com ou sem cobertura ou recheio; | Biscuits, cookies or Graham crackers, with and without toppings or fillings; |
| 17 | Cereal matinal, incluindo aveia em flocos, granola e cereais tipo sucrilhos e all-bran ou nesfit, com ou sem adição de leite, acucar ou adocante; | Breakfast cereal, including ready-to-eat and cooked varieties, such as oats, granola, “Corn Flakes”, “All-Bran” or “Nesfit”, consumed with and without milk, sugar, honey, or artificial sweetener; |
| 18 | Mingau, canjica de milho amarelo (curau), canjica de milho branco (mungunzá); | Porridge, yellow corn pudding (Brazilian ‘*curau”*), white corn pudding (Brazilian “*mungunzá”*); |
| 19 | Frutas frescas, incluindo salada de frutas, banana, laranja ou tangerina / mexerica / bergamota, maçã, mamão, manga, melancia ou melão, acai (podendo ser comprado pronto ou feito a partir da polpa ou fruta) ou qualquer outra fruta; | Fresh fruits, including fruit salad, banana, orange or tangerine/mandarin, apple, papaya, mango, watermelon or cantaloupe, acai (either ready-to-eat or homemade from fruit or pulp), and any other fruit; |
| 20 | Frutas secas, amendoim, nozes ou castanhas, incluindo versões com sal, com açúcar ou pura; | Dried fruits, peanuts, nuts or chestnuts, including salted, sugared or plain versions. |
| 21 | Barra de cereal; | Cereal bar; |
| 22 | Sopas, incluindo preparações caseiras ou versões prontas (congeladas ou enlatadas) ou instantâneas (pacotes). Inclui canja, sopa de legumes com ou sem macarrão e carne, caldo verde, sopa de feijão, lentilha ou ervilha (com ou sem macarrão ou carne), tacacá ou qualquer outro tipo de sopa; | Soups, including homemade, ready-to-eat (frozen or canned) and instant (packaged) varieties. It includes chicken soup, vegetable soup (with and without noodles and meat), green broth, bean, lentil, or pea soup (with and without noodles or meat), ”*tacacá”^^[[2]](#footnote-2)^^* , and any other type of soup; |
| 23 | Macarrão instantâneo; | Instant noodles; |
| 24 | Macarrão sem recheio, do tipo espaguete, talharim, penne ou outro tipo, incluindo preparações caseiras ou versões compradas como refeição pronta congelada com marca. Opções de molho para o macarrão incluem molho de tomate simples, molho bolonhesa, molho branco ou quatro queijos ou qualquer outro tipo de molho, podendo o molho ter sido preparado em casa/restaurante ou comprado pronto em caixinha ou sachê; | Pasta, such as spaghetti, fettuccine, penne, or other types, including homemade or branded ready-to-eat/heat meals. Sauce options include simple tomato sauce, Bolognese sauce, bechamel or cheese sauce, and any other type of sauce. Sauces can be homemade or ready-to-eat or heat, such as tetra pack or sachet versions; |
| 25 | Lasanha, incluindo preparações caseiras ou versões compradas como refeição pronta congelada com marca. Opções de sabor incluem de carne, de presunto, de legumes ou mista; | Lasagna, including homemade or branded ready-to-eat/heat meals, with a variety of sauces and fillings ( meat, ham, vegetable, or mixed); |
| 26 | Macarrão com recheio, ravioli, canelone, capeletti ou outro tipo, incluindo preparações caseiras ou versões compradas como refeição pronta congelada com marca. Opções de molho para o macarrão incluem molho de tomate simples, molho bolonhesa, molho branco ou quatro queijos ou qualquer outro tipo de molho, podendo o molho ter sido preparado em casa/restaurante ou comprado pronto em caixinha ou sachê; | Filled (or stuffed) pasta, such as ravioli, cannelloni, capeletti, and other types, including homemade or branded ready-to-eat/heat meals. Sauce varieties include classic tomato sauce, Bolognese sauce, Alfredo or cheese sauce, and any other type of sauce. Sauces can be homemade or ready-to-eat or heat, bag-in-box and pouch packed; |
| 27 | Torta salgada ou empadão, incluindo preparações caseiras ou versões compradas como refeição pronta congelada com marca. Opções de sabor incluem de frango, de carne, de palmito ou outros legumes, e de queijo; | Savory pie recipes, including homemade or branded ready-to-eat/heat varieties. Filling options include chicken, meat, palm heart or other vegetables, and cheese; |
| 28 | Pizza, incluindo preparações caseiras ou de restaurante ou versões compradas como refeição pronta congelada com marca. Opções de sabor incluem mussarela, marguerita, calabresa, pepperoni, portuguesa, presunto, bacon ou qualquer outro sabor (por exemplo, lombo, frango, quatro queijos, rúcula, vegetariana etc.); | Pizza, including homemade or branded ready-to-eat/heat varieties. Pizza toppings include mozzarella, margherita, Calabrese, pepperoni, Portuguese, ham, bacon, or any other flavor (such as pork loin, chicken, four-cheese, arugula, vegetarian, etc.); |
| 29 | Feijão, lentilha ou grão de bico, podendo ser com ou sem carne, incluindo preparações mistas como feijoada ou baião de dois; | Beans, lentils, or chickpeas, with and without meat, including mixed dishes such as feijoada and “*baiao de dois*”^^[[3]](#footnote-3)^^; |
| 30 | Arroz, incluindo arroz puro ou preparações a base de arroz, como risoto, podendo ser branco ou integral; | Rice, including plain rice and rice-based preparations such as risotto. Rice may be white or whole grain; |
| 31 | Batata frita, podendo ter sido feita a partir de batata crua ou de batata pré-pronta congelada; | French fries, which may have been homemade from scratch or prepared with frozen potatoes; |
| 32 | Batata inglesa ou batata doce, podendo ter sido consumida na forma de purê ou outras preparacoes; | White potatoes or sweet potatoes, including mashed potatoes and other recipes; |
| 33 | Mandioca/aipim, cará ou inhame, podendo ter sido consumidos ser cozido, frito, na forma de purê ou outras preparações; | Cassava/yucca, taro or yam, which may have been consumed boiled, fried, mashed or as part of other recipes; |
| 34 | Farinha, pirão ou farofa de mandioca, podendo a farofa ter sido preparada em casa/restaurante ou comprada pronta de pacote; | Cassava flour, “pirão”^^[[4]](#footnote-4)^^ , or farofa, which may have been prepared at home/restaurant or purchased ready-to-eat.; |
| 35 | Milho verde na espiga ou em lata; | Corn on the cob or canned corn; |
| 36 | Farinha ou farofa de milho, polenta, creme de milho, cuscuz paulista ou marroquino, pamonha salgada ou doce; | Corn flour or “*farofa”*^^[[5]](#footnote-5)^^, polenta, creamed corn, “*paulista couscous*”^^[[6]](#footnote-6)^^ , Moroccan couscous, savory or sweet “*pamonha*”^^[[7]](#footnote-7)^^ ; |
| 37 | Verduras de folha, incluindo alface, acelga, agrião, rúcula, couve, repolho, espinafre, almeirão, escarola, chicória ou outras; | Dark-green leafy vegetables, including lettuce, Swiss chard, watercress, arugula, kale, cabbage, spinach, endive, escarole, chicory, and others; |
| 38 | Legumes, incluindo tomate, pepino, cebola, abóbora, abobrinha, berinjela, cenoura, beterraba, brócolis, couve-flor, vagem, chuchu, jiló, quiabo, maxixe, pimentão, rabanete ou outros legumes; | Vegetables, including tomato, cucumber, onion, pumpkin, zucchini, eggplant, carrot, beetroot, broccoli, cauliflower, green beans, chayote, bitter melon, okra, sweet pepper, radish, or other vegetables. These include all fresh, frozen, and dried options in cooked or raw forms. |
| 39 | Palmito fresco ou em conserva, cogumelo fresco ou em conserva e ervilha em conserva; | Fresh or canned palm heart, fresh or canned mushrooms, and canned peas; |
| 40 | Soja em grão ou produtos à base de soja ou de proteína de soja, incluindo tofu, bife de hamburguer, bolinho ou outras preparações; | Soybeans or processed soy products and soy protein-based products, including tofu, steak and hamburger, meatballs; |
| 41 | Hambúrguer, "Nuggets" ou filé de frango empanado comprados congelados ou consumidos em restaurante fast-food; | Hamburger, chicken nuggets, and chicken tenders which may have been purchased in their branded or frozen forms or consumed at fast-food restaurants; |
| 42 | Carne seca, charque ou jabá, toucinho, torresmo ou bacon, linguiça ou salsicha; | Dried beef, jerked beef, or salted meat, bacon, pork rinds, sausage and “*linguica*”^^[[8]](#footnote-8)^^; |
| 43 | Carnes frescas em geral, diferentes cortes de boi, porco, aves ou miúdos. As carnes podem ter sido consumidas cruas/marinadas, cozidas, refogadas ou ensopadas, assadas, grelhadas, na brasa ou como churrasco, fritas, empanadas ou a milanesa, ou em outra forma de preparação; | Fresh meats in general, including different cuts of beef, pork, poultry, and offal. Meats may have been raw/marinated, cooked, sautéed or stewed, roasted, grilled, barbecued, fried, breaded or prepared in forms; |
| 44 | Sushi, sashimi ou temaki; | Sushi, sashimi or temaki; |
| 45 | Peixes em geral, incluindo salmão, atum ou sardinha, pescada, tilápia (Saint Peter), merluza ou outros peixes, bacalhau ou outro peixe salgado ou peixe enlatado. Os peixes frescos ou congelados podem ser ter sido consumidos crus/marinados, cozidos, refogados ou ensopados, assados, grelhados, na brasa ou como churrasco, fritos, empanados ou a milanesa, ou em outra forma de preparação; | Fish in general, including fresh fish such as salmon, tuna, sardine, hake and tilapia, and fish that has been cured, or preserved, with salt, such as salted cod or canned fish. Fish may have been raw/marinated, cooked, sautéed or stewed, roasted, grilled, barbecued, fried, breaded or prepared in other forms; |
| 46 | Frutos do mar em geral, incluindo camarão fresco, seco ou salgado, caranguejo ou siri, lula ou polvo. Os frutos do mar podem ser ter sido consumidos crus/marinados, cozidos, refogados ou ensopados, assados, grelhados, na brasa ou como churrasco, fritos, empanados ou a milanesa, ou em outra forma de preparação; | Seafood in general, including fresh, dried and salted shrimp, crab, squid and octopus. Seafood may have been raw/marinated, cooked, sautéed or stewed, roasted, grilled, barbecued, fried, breaded or prepared in other forms; |
| 47 | Presunto, mortadela, salame, peito de peru ou similares; | Ham, mortadella, salami, turkey breast or other luncheon meats; |
| 48 | Ovos de galinha ou de outra ave, incluindo ovo frito, mexido, cozido, na forma de omelete ou outra preparação à base de ovo; | Chicken or other bird eggs, including fried, scrambled, boiled and eggs consumed as omelet or in other egg-based preparations; |
| 49 | Queijos, incluindo mussarela ou prato, queijo fresco, ricota ou cottage, meia-cura, curado ou coalho, ralado ou outros tipos de queijo; | All types of cheese, including mozzarella, fresh cheese, ricotta, cottage cheese, semi-cured cheese, cured cheese, rennet cheese, grated cheese, and other types of cheese; |
| 50 | Pipoca doce ou salgada, podendo ter sida feita na hora a partir do grão de milho ou pipoca industrializada (de micro-ondas); | Sweet or salty popcorn, which may be homemade popcorn (made from scratch using popcorn kernels) or industrialized popcorn (such as packaged or microwave popcorn); |
| 51 | Salgados fritos ou assados, incluindo pastel, coxinha, empada, esfirra, quibe, acarajé, abará e outros salgados. Os salgados podem ter sido comprados prontos congelados, preparados em casa ou comprados prontos em restaurante, padaria ou lanchonete; | Fried or baked savory snacks, including “*pastel*”^^[[9]](#footnote-9)^^ “*coxinha*”^^[[10]](#footnote-10)^^ , patty, esfiha, kibbeh, “*acarajé*” or “*abará*”^^[[11]](#footnote-11)^^ and other savory snacks. Snacks may be homemade or purchased from full-service restaurant meals, ready-to-eat from grocery stores, or non-ready-to-eat from grocery stores; |
| 52 | Salgadinho de pacote ou batata chips/palha, incluindo do tipo Cheetos, Doritos, Elma Chips, Ruffles ou outro salgadinho de pacote; | Packaged chips or potato chips/straws, including “Cheetos”, “Doritos”, “Elma Chips”, “Ruffles” and other packaged chips; |
| 53 | Molhos em geral, incluindo ketchup ou barbecue, maionese, mostarda, shoyo ou molho de salada; | Dressings in general, including ketchup or barbecue sauce, mayonnaise, mustard, soy sauce and salad dressings; |
| 54 | Bolo simples ou com cobertura e/ou recheio, torta doce, panetone ou cuca. O alimento pode ter sido comprado pronto sem marca, comprado pronto com marca (do tipo Bauducco, Pullman, Ana Maria etc.), feito em casa a partir de mistura em pó ou feito em casa com farinha e outros ingredientes; | Cake with and without icing and/or filling, pies, panettone or “*cuca*”^^[[12]](#footnote-12)^^ . These include homemade (from cake mix or made from scratch using flour, eggs and other culinary ingredients), or purchased ready-to-eat from grocery stores (such as Bauducco, Pullman, Ana Maria, and other brands) or non-ready-to-eat from grocery stores; |
| 55 | Sorvete, picolé ou geladinho/sacolé; | Ice cream, popsicle or ice pop; |
| 56 | Sobremesas em geral, incluindo arroz doce, cocada, pudim, manjar ou mousse, brigadeiro, gelatina, doce de leite, compotas de frutas, goiabada, doce de abóbora ou marmelada, ou outra sobremesa. A sobremesa pode ter sido comprada pronta sem marca (por exemplo, em padaria, feiras etc.), comprada pronta com marca, feita em casa a partir de mistura pronta ou feita em casa a partir de ingredientes; | Desserts in general, including rice pudding, coconut sweet, pudding, “*manjar*”^^[[13]](#footnote-13)^^ or mousse, “*brigadeiro*”^^[[14]](#footnote-14)^^ , gelatin, fudge, fruit preserves, guava paste, pumpkin or quince sweet, and other types of dessert. These include homemade desserts (from dessert mix or made from scratch), or purchased ready-to-eat from grocery stores (such as Bauducco, Pullman, Ana Maria, and other brands) or non-ready-to-eat from grocery stores; |
| 57 | Chocolate, bombom ou trufa, bala, caramelo, pirulito ou chiclete, pé de moleque, paçoca ou doce de amendoim, algodão doce ou outra guloseima. | Chocolate bars or candies, caramel or other types of candies, lollipop or chewing gum, peanut brittle, “*paçoca*”^^[[15]](#footnote-15)^^ or peanut candy, cotton candy, and other sweets treats. |

**Supplementary Table S2. Comparison of dietary energy contribution of Nova food groups and subgroups estimated from the Nova24h and an interviewer-led 24 h recall (n=186)**

|  |  | % of total energy intake | | | | | | |  | Intraclass correlation coefficients | | |
| --- | --- | --- | --- | --- | --- | --- | --- | --- | --- | --- | --- | --- |
| Nova food groups and subgroups |  | Nova24h | | |  | Reference tool | | |  |  |  |  |
|  |  | Mean | 95% CI | |  | Mean | 95% CI | |  | ICC | 95% CI | |
| **Unprocessed or minimally processed foods** |  | **52.3** | **49.9** | **54.7** |  | **52.6** | **50.0** | **55.3** |  | **0.78** | **0.71** | **0.84** |
| Red meat |  | 8.4 | 6.8 | 10.0 |  | 7.0 | 5.5 | 8.5 |  | 0.83 | 0.77 | 0.87 |
| Milk and plain yogurt^1^ |  | 4.6 | 3.7 | 5.4 |  | 6.5 | 4.9 | 8.1 |  | 0.50 | 0.33 | 0.62 |
| Fruit |  | 7.8 | 6.7 | 8.8 |  | 5.7 | 4.8 | 6.6 |  | 0.78 | 0.67 | 0.85 |
| Grains |  | 5.5 | 4.8 | 6.3 |  | 5.4 | 4.4 | 6.4 |  | 0.67 | 0.56 | 0.75 |
| Legumes |  | 4.2 | 3.5 | 4.9 |  | 4.6 | 3.8 | 5.5 |  | 0.74 | 0.65 | 0.81 |
| Poultry |  | 4.0 | 3.1 | 4.9 |  | 3.9 | 3.0 | 4.8 |  | 0.76 | 0.68 | 0.82 |
| Eggs |  | 3.0 | 2.4 | 3.6 |  | 3.0 | 2.2 | 3.7 |  | 0.88 | 0.84 | 0.91 |
| Vegetables |  | 1.7 | 1.5 | 1.9 |  | 2.7 | 2.2 | 3.1 |  | 0.54 | 0.36 | 0.66 |
| Flours^2^ |  | 2.2 | 1.6 | 2.7 |  | 2.4 | 1.6 | 3.2 |  | 0.31 | 0.08 | 0.49 |
| Roots and tubers |  | 1.9 | 1.4 | 2.3 |  | 2.4 | 1.6 | 3.1 |  | 0.62 | 0.49 | 0.71 |
| Freshly squeezed fruit juices^3^ |  | 2.4 | 1.8 | 3.0 |  | 2.3 | 1.7 | 3.0 |  | 0.66 | 0.54 | 0.74 |
| Pasta |  | 2.5 | 1.6 | 3.4 |  | 2.0 | 1.2 | 2.7 |  | 0.85 | 0.80 | 0.89 |
| Fish and seafood |  | 1.5 | 0.8 | 2.2 |  | 1.2 | 0.6 | 1.8 |  | 0.80 | 0.73 | 0.85 |
| Nuts and seeds without salt, sugar, or oil |  | 1.2 | 0.8 | 1.7 |  | 1.1 | 0.5 | 1.5 |  | 0.69 | 0.59 | 0.77 |
| Coffee and tea |  | 0.6 | 0.5 | 0.6 |  | 0.9 | 0.8 | 1.1 |  | 0.50 | 0.31 | 0.64 |
| Other unprocessed or minimally processed foods^4^ |  | 0.8 | 0.3 | 1.2 |  | 1.5 | 0.9 | 2.1 |  | 0.43 | 0.24 | 0.57 |
| **Processed culinary ingredients** |  | **11.6** | **10.4** | **12.8** |  | **11.9** | **10.6** | **13.2** |  | **0.54** | **0.38** | **0.65** |
| Plant oils |  | 3.9 | 3.5 | 4.3 |  | 6.0 | 5.2 | 6.7 |  | 0.31 | 0.08 | 0.48 |
| Sugar, honey, or molasses |  | 4.2 | 3.5 | 4.8 |  | 2.9 | 2.2 | 3.6 |  | 0.67 | 0.55 | 0.75 |
| Animal fats^5^ |  | 1.8 | 1.4 | 2.3 |  | 2.0 | 1.3 | 2.7 |  | 0.65 | 0.53 | 0.74 |
| Other processed culinary ingredients^6^ |  | 1.7 | 1.1 | 2.3 |  | 1.0 | 0.4 | 1.5 |  | 0.58 | 0.44 | 0.68 |
| **Processed foods** |  | **17.1** | **15.2** | **19.0** |  | **14.7** | **12.5** | **16.5** |  | **0.72** | **0.62** | **0.79** |
| Cheese |  | 6.6 | 5.3 | 7.8 |  | 4.7 | 3.6 | 5.6 |  | 0.67 | 0.55 | 0.76 |
| Processed bread |  | 3.9 | 3.0 | 4.8 |  | 4.1 | 3.1 | 5.0 |  | 0.78 | 0.70 | 0.83 |
| Wine and beer |  | 2.9 | 1.8 | 4.0 |  | 2.4 | 1.2 | 3.6 |  | 0.84 | 0.78 | 0.88 |
| Processed cakes and desserts^7^ |  | 1.7 | 1.0 | 2.4 |  | 1.1 | 0.5 | 1.5 |  | 0.55 | 0.40 | 0.66 |
| Ham and other salted, smoked, or canned meat or fish |  | 0.8 | 0.4 | 1.2 |  | 0.9 | 0.5 | 1.4 |  | 0.70 | 0.61 | 0.78 |
| Nuts and seeds with salt or sugar |  | 0.5 | 0.2 | 0.9 |  | 0.8 | 0.1 | 1.5 |  | 0.47 | 0.30 | 0.61 |
| Savory snacks, including croquettes, pastries, and mini pies^8^ |  | 0.5 | 0.0 | 1.0 |  | 0.3 | 0.0 | 0.6 |  | 0.68 | 0.57 | 0.76 |
| Other processed foods^9^ |  | 0.2 | 0.1 | 0.3 |  | 0.5 | 0.3 | 0.8 |  | 0.20 | -0.06 | 0.40 |
| **Ultra-processed foods** |  | **19.0** | **17.0** | **20.9** |  | **20.8** | **18.5** | **23.2** |  | **0.75** | **0.66** | **0.81** |
| Cakes, cookies, and pies |  | 1.6 | 1.0 | 2.1 |  | 2.9 | 1.8 | 3.9 |  | 0.63 | 0.51 | 0.73 |
| Breads^10^ |  | 2.5 | 1.8 | 3.2 |  | 2.8 | 2.0 | 3.7 |  | 0.70 | 0.59 | 0.77 |
| Desserts and sweets^11^ |  | 2.6 | 1.9 | 3.3 |  | 2.8 | 1.9 | 3.6 |  | 0.72 | 0.63 | 0.79 |
| Reconstituted meat or fish products |  | 2.6 | 1.8 | 3.3 |  | 2.2 | 1.5 | 2.8 |  | 0.52 | 0.36 | 0.64 |
| Ice cream and ice pops |  | 1.8 | 1.0 | 2.5 |  | 1.3 | 0.6 | 2.0 |  | 0.82 | 0.76 | 0.86 |
| Crackers |  | 0.8 | 0.6 | 1.1 |  | 1.2 | 0.6 | 1.8 |  | 0.54 | 0.39 | 0.66 |
| Soft drinks |  | 1.1 | 0.6 | 1.6 |  | 1.2 | 0.7 | 1.7 |  | 0.83 | 0.78 | 0.88 |
| Milk-based drinks and flavored yogurts |  | 0.8 | 0.5 | 1.0 |  | 1.0 | 0.7 | 1.4 |  | 0.55 | 0.40 | 0.66 |
| Sauces, dressings, and gravies |  | 0.7 | 0.4 | 0.9 |  | 0.8 | 0.4 | 1.2 |  | 0.60 | 0.47 | 0.70 |
| Breakfast cereals |  | 0.5 | 0.3 | 0.7 |  | 0.8 | 0.3 | 1.2 |  | 0.54 | 0.38 | 0.65 |
| Margarine |  | 0.3 | 0.1 | 0.5 |  | 0.6 | 0.3 | 0.9 |  | 0.54 | 0.38 | 0.65 |
| Salty snacks |  | 0.4 | 0.1 | 0.6 |  | 0.3 | 0.0 | 0.6 |  | 0.78 | 0.71 | 0.83 |
| Instant and canned soups |  | 0.2 | 0.0 | 0.4 |  | 0.1 | 0.0 | 0.3 |  | 0.79 | 0.72 | 0.84 |
| Other ultra-processed foods^12^ |  | 3.1 | 2.1 | 4.1 |  | 2.8 | 1.9 | 3.6 |  | 0.30 | 0.07 | 0.48 |
| **Total** |  | **100.0** |  |  |  | **100.0** |  |  |  |  |  |  |

ICC, Intraclass correlation coefficients

^1^Includes milk consumed with coffee, fruit, and powdered chocolate

^2^Includes wheat flour, cassava flour and corn flour

^3^Includes juice from fruit pulp

^4^Includes a few homemade pastries, sweet or savory

^5^Includes salted and unsalted butter

^6^Includes textured soy protein, baking powder; vinegar; cornstarch

^7^Includes processed cakes and desserts (store-bought, prepacked for direct sale such as from deli counters)

^8^Includes deep-fried croquettes or pastries with different fillings (cheese, chicken, beef, ham, and others), and mini pies with a filling

^9^Includes processed juices and fruits and veggies preserved in brine

^10^Includes industrial-processed breads and buns

^11^Includes chocolate bars, candies, cereal bars and gelatin desserts, puddings, and *brigadeiro* (dessert made of condensed milk and cocoa powder)

^12^Includes soy protein-based drinks, fruit juices and energy drinks; other sweetened drinks; distilled alcoholic drinks; sweeteners; spread cheese; microwavable popcorn; frozen meals and ready-to-eat/heat pizza, savory pies, and French fries

1. Traditional Brazilian biscuit made with a base of corn flour. [↑](#footnote-ref-1)
2. A typical Brazilian soup made with dried shrimps, tucupí (wild cassava byproduct), alfavaca (wild Amazonian basil), manioc starch, hot yellow peppers, and jambú – a leafy plant with anesthetic properties. [↑](#footnote-ref-2)
3. A typical Brazilian recipe made with a base of rice and beans. [↑](#footnote-ref-3)
4. A typical Brazilian recipe made with a base of cassava flour. [↑](#footnote-ref-4)
5. Buttery and toasty Brazilian side dish made with cassava flour. [↑](#footnote-ref-5)
6. A typical Brazilian recipe made with a base of corn and cassava flour. [↑](#footnote-ref-6)
7. A typical Brazilian recipe made with a base of corn flour. [↑](#footnote-ref-7)
8. Brazilian-style smoked sausage. [↑](#footnote-ref-8)
9. A Brazilian style of fried pastry with fillings. [↑](#footnote-ref-9)
10. A Brazilian style of breaded pastry with a chicken filling. [↑](#footnote-ref-10)
11. Acarajé and abará are typical Brazilian dishes made with black-eyed peas filled with shrimp. Acarajé is shaped into balls and deep-fried in boiling *azeite de dende* also known as Brazilian palm oil. [↑](#footnote-ref-11)
12. A typical Brazilian cake with fruits or other fillings. [↑](#footnote-ref-12)
13. A typical Brazilian pudding made with a base of coconut. [↑](#footnote-ref-13)
14. A typical Brazilian dessert made of condensed milk, cocoa powder, butter, and chocolate sprinkles covering the outside layer. [↑](#footnote-ref-14)
15. A typical Brazilian peanut based sweet. [↑](#footnote-ref-15)
